# Social participation and cognitive activities as explanation factor for the association between income and self-rated health for older adults

**DOI:** 10.3389/fpubh.2024.1435945

**Published:** 2024-11-28

**Authors:** A. M. Buß, F. G. Wittmann, A. Pabst, M. Luppa, A. Oey, I. Blotenberg, M. I. Cardona, S. Weise, A. Bauer, R. P. Kosilek, F. Bader, C. Brettschneider, B. Wiese, W. Hoffmann, T. Frese, J. S. Gensichen, H.-H. König, J. R. Thyrian, S. G. Riedel-Heller

**Affiliations:** ^1^Institute of Social Medicine, Occupational Health and Public Health (ISAP), University of Leipzig, Leipzig, Germany; ^2^Institute for General Practice, Work Group Medical Statistics and IT-Infrastructure, Hannover Medical School, Hannover, Germany; ^3^German Center for Neurodegenerative Diseases (DZNE), Site Rostock/Greifswald, Greifswald, Germany; ^4^Institute of General Practice and Family Medicine, Martin-Luther-University Halle-Wittenberg, Halle, Germany; ^5^Institute of General Practice and Family Medicine, LMU University Hospital, Munich, Germany; ^6^Department of Health Economics and Health Services Research, University Medical Centre Hamburg-Eppendorf, Hamburg, Germany; ^7^Institute for Community Medicine, University Medicine Greifswald (UMG), University of Greifswald, Greifswald, Germany; ^8^Faculty V: School of Life Sciences, University of Siegen, Siegen, Germany

**Keywords:** income, self-rated health, fruit and vegetable consumption, social participation, physical activities, cognitive activities, higher age groups

## Abstract

**Introduction:**

Health disparities pose a considerable challenge for older adults individuals, particularly those with a heightened risk of developing dementia. Discrepancies in health status among various income brackets are only partially attributable to structural factors such as working and living conditions or the quality of food. The aim of this study was therefore to explore whether and to what extent various health-promoting behaviors can explain the association between household income and self-rated health among older people at risk of dementia.

**Methods:**

The sample consisted of 845 participants (average age 68.9 years; 52.6% female) from the AgeWell.de study, an intervention trial aiming to preserve cognitive function. The participants exhibited an increased risk of dementia, defined by a CAIDE (Cardiovascular Risk Factors, Aging and Dementia) score of at least nine points. To explore the relationship between household income and self-rated health, measured using the EQ-5D-VAS, a regression analysis was conducted. This association was then examined using four mediation analyses that included health-promoting behaviors such as fruit and vegetable consumption, social participation, physical activities, and cognitive activities.

**Results:**

The results reveal a positive association between higher income and self-rated health. This relationship is mediated by social participation. Additionally, cognitive activities were found to partially mediate this correlation. Neither physical activities nor fruit and vegetable consumption could account for the association between income and self-rated health.

**Conclusion:**

The findings have the potential to advance research on the correlation between income and health among older age cohorts at risk of developing dementia. They highlight the potential significance of social engagement and cognitive activities for health and may inspire the development of strategies aimed at enhancing accessibility to activities such as cultural events, educational institutions, and participation in courses for a wider audience.

## Introduction

General health has improved over the past decades in all countries for which data are available (1). However, this development appears to progress slowest in socially disadvantaged groups (2). It was found that there are internationally consistent differences in morbidity, mortality, and life expectancy among social strata, with individuals from lower status groups being disadvantaged (3). Higher education and elevated occupational status can positively influence health, as individuals with higher education tend to possess greater knowledge about health promotion, while those in higher-skilled positions often experience lower levels of workplace stress (4). Similarly, income disparities can have a significant impact on various aspects of life and consequently on dimensions of health. A low income is associated with various restrictions and burdens in everyday life, such as precarious living conditions, unhealthy working environments, and limited access to material resources, necessities and consumer goods (5, 6). All these factors, in turn, have a negative impact on mental and physical health, and lastly on mortality (5, 7, 8). Therefore, income serves as an important tool in assessing health inequalities. Particularly for the health of individuals aged 60 and above, income has been identified as a significant indicator of social status compared to education and occupation (9). For older adults at heightened risk of developing dementia, the relationship between income and health may play a particularly relevant role. After all, they face significant challenges including limited mobility, social isolation, and the risk of cognitive decline (10). Several studies have already found an association between lower income and higher dementia prevalence (11, 12). Therefore, this demographic may stand to benefit particularly from adequate healthcare and support in adopting health-promoting behaviors.

Bartley (13) argues, that the effect of income on health, as evidenced by numerous studies, cannot solely be attributed to factors such as living and working conditions or the quality of food. In addition to these structural conditions, health-promoting behaviors can also be considered (5). However, health behavior should not be perceived as individualized, but also as a product of structural inequalities. Subjective behavioral decisions can be viewed against the backdrop of structural conditions, such as societal positioning within the social hierarchy. Income, which is unevenly distributed across social strata, serves as an important indicator of this positioning within the hierarchical structure. In Germany, previous studies on the topic of health behavior and social inequality have mostly focused on the relationship between socioeconomic status and health-promoting behaviors. However, since this status includes not only income but also the dimensions of education and occupation, examining the relationship between income and health behavior can provide additional insights.

Self-rated health is a common measure of health status (14), explicitly recommended by the WHO (15). This is due in part to its outstanding prognostic properties (14). Self-rated health has the advantage over other health measurement instruments in that it can take into account many different factors (16). In addition to well-known and easily ascertainable aspects such as diagnoses or physical or mental health symptoms, latent characteristics such as the subjective severity and impact of health problems can also be captured (17). It has been shown to be an appropriate measure of health status, particularly for older age groups (*ibid.*). Against this background, this study addressed the following research question:

*To what extent do various forms of health-promoting behaviors influence the relationship between income and self-rated health among older people at risk of developing dementia?*

Therefore, this study examines the influence of the following health-related behaviors, chosen for their established links to cognitive decline in older adults (18–21), which are also amenable to modification within the context of a prevention. It is expected that a stronger engagement in these behaviors will lead to higher self-rated health. One aspect to be examined is the impact of a healthy diet measured through fruit and vegetable consumption on self-rated health. The German Nutrition Society (DGE) recommends that adults consume at least 400 grams of vegetables and 250 grams of fruit daily, which is approximately equivalent to five servings of fruits and vegetables (22). Studies have shown that higher income is associated with increased consumption of fruits and vegetables (23–25). It has been found that poor nutrition and below-average physical activity are associated with obesity, hypertension, and diabetes mellitus (26, 27). These factors, in turn, contribute to the onset of dementia (18) and are associated with an increased risk of cardiovascular diseases such as heart attack or failure, tumors, and respiratory diseases (28).

A second factor to be investigated for its influence on the relationship between income and health concerns social participation. Social participation, in the context of this study, encompasses both social activities in private settings with family and friends, as well as integration into societal life and cultural events. Research has shown that there is an association between income poverty and social integration in societal life as well as cultural participation opportunities (5, 29, 30). Furthermore, engagement in social participation opportunities appears to decline with increasing age (31). However, they assume a particularly significant role in older age, as social contacts and participation in public life are associated not only with higher life satisfaction and lower levels of depression (19) but also with a reduced risk of developing dementia (32, 33). Moreover, Lindstrom and colleagues found in a case–control study that social activities, such as membership in social or religious organizations, visits with friends, or phone calls, have a reducing effect on the likelihood of developing Alzheimer’s disease (33).

Subsequently, the influence of physical activity on the relationship between income and self-rated health is being explored. Physical activity includes all forms of bodily exertion in daily life, whether through intentional sport or as a side effect of everyday activities. Various studies have found that each the frequency and the duration of engaging in physical activities varies according to income group and diminishes with advancing age (34–36). Physical activities, in turn, are associated with reducing various health risks (18, 26–28) and have a positive effect on cognitive function. Twenty-two physical activity intervention studies showed an effect on the cognitive function of persons with dementia, 0.4803 (95% CI = 0.1901–0.7704), with a high percentage of heterogeneity (*I*^2^ = 86%, *p* ≤ 0.0001) (*ibid.*). Furthermore, Netz and colleagues (37) provide an overview of the evidence regarding the effects of physical activities on the mental health of older adults based on their meta-analysis, in which they compiled the results of 36 intervention studies. They noted that there was a significant association between the overall effect of intervention groups engaging in physical activity over several weeks and well-being compared to control groups (*ibid.*).

Finally, the influence of cognitive activities, defined as mentally stimulating endeavors such as acquiring new skills, solving puzzles, and reading, on the relationship between income and self-rated health was examined. Studies have found a positive correlation between the financial status and the frequency of engaging in cognitively stimulating activities (38). For older age groups, engaging in cognitive activities appears to be particularly important, as it has been found to be associated with improved cognitive performance (20) and a reduced likelihood of developing Alzheimer’s disease (33).

## Methods

### Study population

The analyses were conducted using the baseline data collected in 2019 from the AgeWell.de study. This is a multicentred, cluster-randomized, controlled multi-component intervention study in primary care, aiming at preventing cognitive decline in older adults through a two-year intervention (39). Overall, 1,030 primary care patients, including 537 (52.1%) women, were recruited at five locations (Greifswald, Halle, Kiel, Leipzig, Munich) from June 2018 to October 2019 (39). The participating general practices were randomized into the intervention and control groups (40). The multi-component intervention included medication management, nutritional counseling, physical activity, cognitive training, social activities, and intervention in case of grief and depression. The control group received general health counseling regarding the intervention components, along with standard care from their general practitioners (*ibid.*). At the time of the baseline assessment, no intervention had yet been implemented; therefore, group assignment will not be considered in the following analysis. Baseline data were collected through personal interviews, during which neuropsychological testing was conducted to assess cognitive performance. Sociodemographic information, along with various health-related aspects and lifestyle factors, was also collected. Additionally, participants were provided with a self-administered questionnaire that solicited detailed information regarding dietary habits and alcohol consumption.

After excluding all cases where at least one item was unanswered, the sample consisted of 845 participants. The inclusion criteria comprised a minimum age of 60 years and an elevated risk for dementia development, as defined by a CAIDE-score of at least 9 points. The exclusion criteria, on the other hand, included diagnosed or suspected dementia by the primary care physicians, severe clinical depression, significant pre-existing medical conditions, severe hearing, visual, communication, or mobility impairments, lack of proficiency in the German language, and participation in another intervention study (39).

### Variables/measures

Health status, the dependent variable, was assessed in this study using self-rated health, measured by the EQ-5D-VAS. This instrument employs a visual analog scale, akin to a thermometer, where participants indicate their current health status (41). The participants estimate their current health on a scale from 0 to 100 points, whereby higher score indicates better self-rated health.

The household income serves as the independent variable, selected for its capacity to accurately depict an individual’s financial standing, particularly considering the common disparity in income distribution among household members. The question is derived from a standardized questionnaire developed by the Institute of Social Medicine, Occupational Medicine, and Public Health (ISAP), Medical Faculty, University of Leipzig. Household income was reported in 100 Euro intervals after deduction of insurance contributions, taxes, and operational expenditures.

The analyses were controlled for age at the beginning of the study, gender, coded dichotomously, as well as education, measured using the CASMIN (Comparative Analysis of Social Mobility in Industrial Nations) classification, which considers various general and vocational qualifications, dividing them into three levels: low, medium, and high (42), and household size.

#### Mediators

The questions regarding fruit and vegetable consumption originate from the German Health Interview and Examination Survey for Adults (DEGS) nutritional questionnaire developed by the Robert Koch Institute (22). To conduct the variable, the following foods were queried: fresh fruit, cooked fruit, fruit juice, fresh vegetables, cooked vegetables, legumes, and vegetable juice. For each form of food, participants were asked how often they consumed it and how many portions they ate on average. For juice intake, they were also asked about the mixing ratio. Subsequently, the values were converted to daily consumption and a composite score was created from the seven items. For the present study, fruit and vegetable consumption was integrated into the model in kilograms.

The items concerning social participation are derived from a standardized questionnaire catalog developed by ISAP (40). This included the following nine items: regular engagement in hobbies with others, such as playing cards, gymnastics, dancing, or similar activities; involvement in a church organization; participation in a club, association, or political party; regular social activities such as going to the cinema, theater, restaurants, or pubs; traveling with others; hiking or cycling tours with others; volunteering activities; attending local adult education centers; and other social activities. One point was assigned for each social activity if it was performed regularly, otherwise, zero points were given. Finally, a total score was calculated from the items, with a maximum of nine points.

The questions regarding physical and cognitive activities are also drawn from a standardized questionnaire catalog developed by ISAP (39). The coding methodology adhered to the framework outlined by Verghese et al. (43), assigning 0 points to the response categories *Never* and *Less than once a week*, 1 point to *Once a week*, 4 points to *Several times a week*, and 7 points to *Every day*. Subsequently, sum scores were computed for both physical and cognitive activities based on these points. The first 10 questions of the questionnaire address physical activities such as cycling; walking; swimming; gymnastics or calisthenics; fitness or strength training; sports such as football, handball, basketball, volleyball, badminton, rowing, martial arts; other sports such as bowling, dancing, stationary cycling, light jogging, golf; pedometer use; house and garden work; and caring for relatives or friends. The remaining 12 questions pertained to cognitive activities: crossword puzzles; memory and cognitive tasks; card and board games, such as chess; social engagement, such as caring for the needy, tutoring, volunteering, in the church community, in a nursing home, political party, or association; learning new things, for example, in a sports, dance, cooking, or language course; reading books, newspapers, or recipes; writing stories, poems, or letters; playing musical instruments, in a choir, or with family; phone calls; mobile phone usage; computer usage; usage of other technical devices such as a video recorder or a DVD player.

The internal consistencies of the three sum scores for social participation (*Cronbach’s α* = 0.64), physical activities (*Cronbach’s α* = 0.40), and cognitive activities (*Cronbach’s α* = 0.57) did not approach the threshold of 0.80 as recommended by Bortz and Döring (44). However, the three indices are each formative measurement models, in which the items, unlike reflective models, are not influenced by a common factor (45) but complement each other substantively. Consequently, it is also not necessary for the items to exhibit clear correlations with each other or to be internally consistent (*ibid.*). Given the utilization of standardized questionnaires in all cases and the complementary nature of the questions in content, no selection process was undertaken to avoid loss of information. Moreover, no substantial improvement in any composite score was observed by removing any item, with a gain exceeding 0.03.

### Statistical analysis

The item *Other social activities* of the variable *Social Participation* generated 222 missing values. Upon closer examination of the data, it seemed plausible that a lack of response to this question should be interpreted as “no other activities,” hence these cases were not excluded from the sample.

Conducting Welch tests revealed that the group of participants differed significantly only in terms of household size from the group with item non-response, *t* = 2.25, *p* < 0.05. The group of those excluded from the analyses lived on average in a larger household than the group of participants. However, the effect size is only 0.2, categorizing it as small according to Cohen’s classification (46).

To gain an overview of the data, initial descriptive analyses were conducted. In the next step, the relationship between income and self-rated health was determined using a regression with bootstrapping. This method was chosen because the Shapiro–Wilk test suggested a violation of normal distribution during the assumption checking process for linear regression, *W* = 0.96, *p* < 0.001. Regression with bootstrapping is a robust procedure and offers the advantage over linear regressions of not making assumptions about the distribution of residuals (47).

Subsequently, the impact of health-related behaviors including fruit and vegetable consumption, social participation, physical, and cognitive activities on the association between income and self-rated health was assessed. For this purpose, four mediation analyses using bootstrapping were conducted. During the assumption checks, Rainbow tests indicated no violation of the linearity assumption. Given the robust procedure chosen, assumption checks concerning the distribution of residuals were deemed unnecessary. Each of the four mediations was computed with 10,000 bootstraps. Covariates including age, gender, education, and household size were controlled for in all five models.

All analyses were conducted using the statistical software R-Studio, Version 1.3.1093. The mediation analyses were performed using the PROCESS extension developed by Andrew F. Hayes (48).

## Results

### Descriptive overview

Table 1 provides a descriptive overview of the results. 50% of the participants rated their health on a scale of 0 to 100 with at least 80 points.

**Table 1:** Sample characteristics (*n* = 845).

	% or mean	Med	SD	Min	Max
Health^1^	76.37	80	15.85	9	100
Income	2155.77	1875	1233.76	375	9,000
Fruits and vegetables	0.53	0.4	0.48	0	4.54
Social participation	3.42	3	1.88	0	9
Physical activities	14.91	14	7.65	0	46
Cognitive activities	32.34	32	11.75	0	71
Age	68.94	69	4.91	60	78
Female gender	52.54%	1	0.5	0	1
Education					
Low	24.14%	0	0.43	0	1
Middle	53.37%	1	0.5	0	1
High	22.49%	0	0.42	0	1
Household size					
1	30.77%	0	0.46	0	1
2	66.04%	1	0.47	0	1
3	2.60%	0	0.16	0	1
4	0.36%	0	0.06	0	1
5	0.12%	0	0.03	0	1
6	0.12%	0	0.03	0	1

% = relative frequency, Med = median, SD = Standard deviation, Min = Minimum, Max = Maximum.

Highest attainable score for social participation: 9, highest attainable score for physical activities: 70, highest attainable score for cognitive activities: 84.

1 Health measured by self-rated health (EQ-5D-VAS), Income values are given in 1-euro increments, based on household income.

The median household income was 1875 euros. Only 26.51% of the participants consumed the recommended minimum amount of 650 grams of fruits and vegetables daily. On average, participants consumed 530 grams of fruits and vegetables per day and regularly took part in three social participation opportunities. Half of the respondents scored a maximum of 14 out of 70 possible points for physical activities and 32 out of 84 points for cognitive activities. At the time of the survey, the mean age of the participants was 69 years, with 52.54% of them being female. The majority of respondents, 53.37%, had a moderate level of education, while 24.14% had a low level and 22.49% had a high level of education. A significant portion of the participants, 66.04%, lived in households with two members, 30.77% lived alone, and 3.19% lived in households with three or more individuals.

### Income and health

Table 2 shows the results of the regression model of the association between income and self-rated health. The results indicate that household income has a small but positive effect on self-rated health, *b* = 0.17, *p* < 0.001. For every increase of 100 euros in income, self-rated health increases by 0.17 points. None of the covariates had a significant effect on health. The model explains approximately 3% of the total variance in self-rated health status and demonstrates a significant model fit, *p* < 0.001.

**Table 2:** Robust regression with bootstrapping to the association between income and self-rated health.

Health^1^				
	ME	β	Lower limit	Upper limit
Income	0.17***	0.13	0.038	0.281
Age	0.13	0.04	−0.087	0.349
Female gender	−0.02	−0.00	−2.210	2.148
Education	1.40	0.06	−0.004	2.950
Household size	1.41	0.05	−1.114	3.905
Bootstraps	10,000			
*R* ^2^	0.03			
Adj. *R*^2^	0.03			

ME, Marginal effects; β, standardized beta coefficients.

*p < 0.05, **p < 0.01, ***p < 0.001.

1 Health measured by self-rated health (EQ-5D-VAS), Income values are given in 100-euro increments, based on household income.

### Influence of health-related behaviors

Table 3 shows the results from the mediation analyses. Fruit and vegetable consumption had no effect on the relationship between income and self-rated health, indirect effect *ab* = 0.00; *95% CI*[−0.004; 0.011]. The results also indicate that women consume more fruits and vegetables than men.

**Table 3:** Mediation analyses.

	Fruits and vegetables	Social participation	Physical activities	Cognitive activities
Income	0.00 (0.00)	0.05 (0.01)***	0.04 (0.02)	0.18 (0.03)***
Age	0.00 (0.00)	0.02 (0.01)	−0.00 (0.05)	−0.19 (0.08)*
Female gender	0.18 (0.03)***	0.13 (0.13)*	2.12 (0.54)***	2.40 (0.77)**
Education	0.02 (0.02)	0.22 (0.10)*	0.92 (0.40)*	4.87 (0.57)***
Household size	0.01 (0.03)	−0.16 (0.12)	0.49 (0.51)	−1.75 (0.72)*
*R* ^2^	0.04	0.10	0.03	0.16
	Health^1^	Health^1^	Health^1^	Health^1^
Income	0.17 (0.05)***	0.09 (0.05)	0.16 (0.05)**	0.15 (0.05)**
Fruits and vegetables	0.93 (1.15)	–	–	–
Social participation	–	1.72 (0.30)***	–	–
Physical activities	–	–	0.38 (0.07)***	–
Cognitive activities	–	–	–	0.12 (0.05)*
Age	0.12 (0.11)	0.09 (0.11)	0.13 (0.11)	0.15 (0.11)
Female gender	−0.19 (0.13)	−0.51 (1.09)	−0.83 (1.10)	−0.32 (1.12)
Education	1.18 (0.83)	1.02 (0.82)	1.05 (0.82)	0.80 (0.86)
Household size	1.39 (1.05)	1.68 (1.03)	1.22 (1.03)	1.62 (1.05)
*R* ^2^	0.03	0.07	0.07	0.04

Marginal effects are reported, followed by standard errors in brackets.

*p < 0.05, **p < 0.01, ***p < 0.001.

1 Health measured by self-rated health (EQ-5D-VAS), Income values are given in 100-euro increments, based on household income.

**Figure 1:**
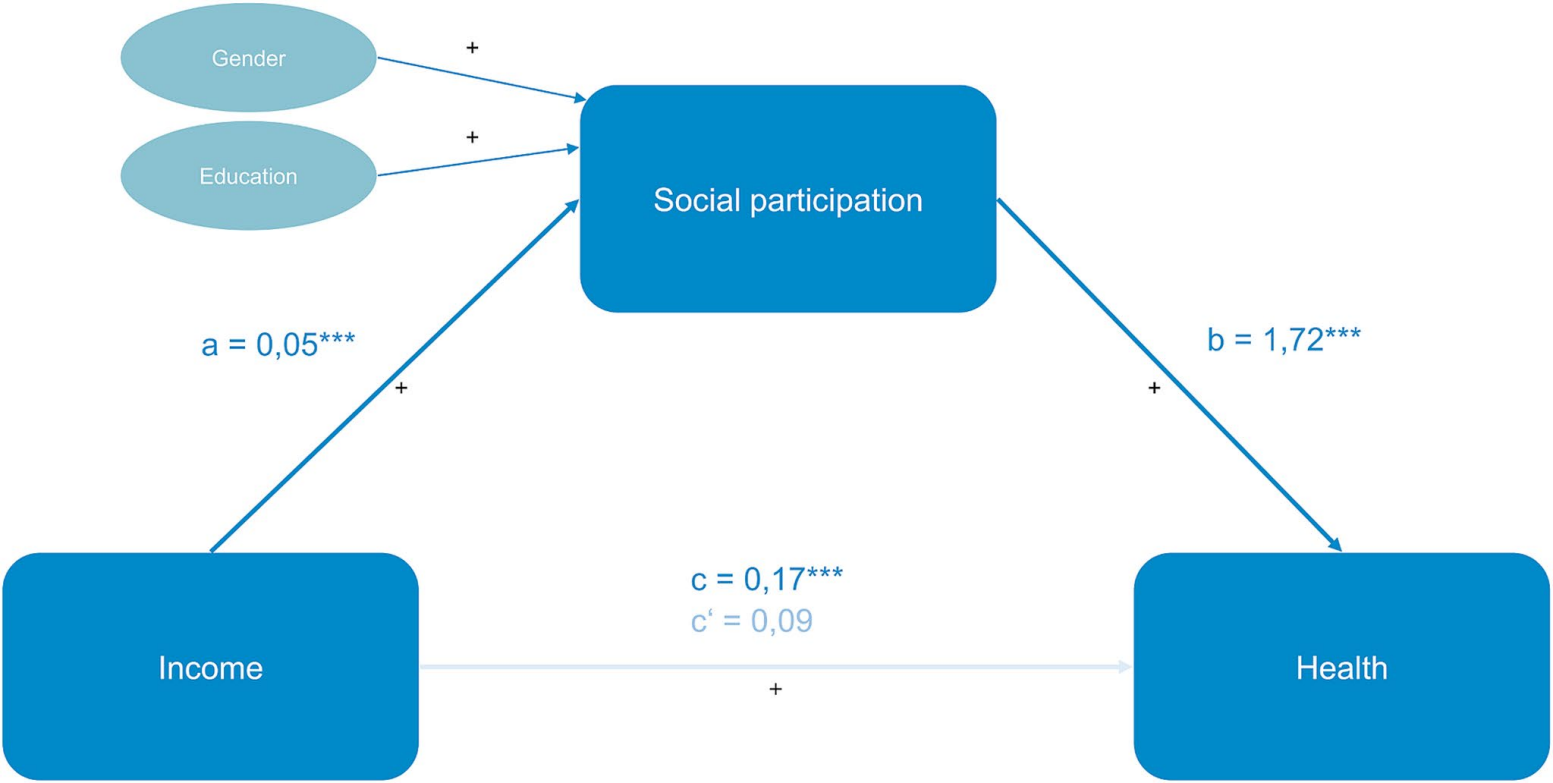
Mediation on household income, social participation, and self-rated health. Significance: *p < 0.05, **p < 0.01, ***p < 0.001. Significant effects are highlighted in green, non-significant in red. + represents a positive effect, − represents a negative effect. c = total effect, c‘= direct effect, a = effect of independent variable on mediator, b = effect of mediator on dependent variable. Only control variables with a significant effect on the mediator variable were included in the figure. Health was measured using self-reported health (self-rated-health, EQ-5D-VAS), income values are given in 100-euro increments based on household income, male gender as reference category.

However, social participation mediates the relationship between income and health, with an indirect effect of *ab* = 0.08; *95% CI*[0.048; 0.114]. The percentage of the total effect mediated is 47%, suggesting that almost half of the overall effect may be explained by social participation. Additionally, the results suggest that women participate significantly more in social activities compared to men, and that social participation increases with higher education levels. For a visual representation of the mediation (see Figure 1).

Physical activities cannot explain the relationship between income and self-rated health, indirect effect *ab* = 0.01; *95% CI*[−0.003; 0.035]. However, they are positive associated with self-rated health, *b* = 0.38, *p* < 0.001. Furthermore, the findings suggest that individuals with higher levels of education and women engage in physical activity significantly more frequently compared to those with lower education levels and men.

**Figure 2:**
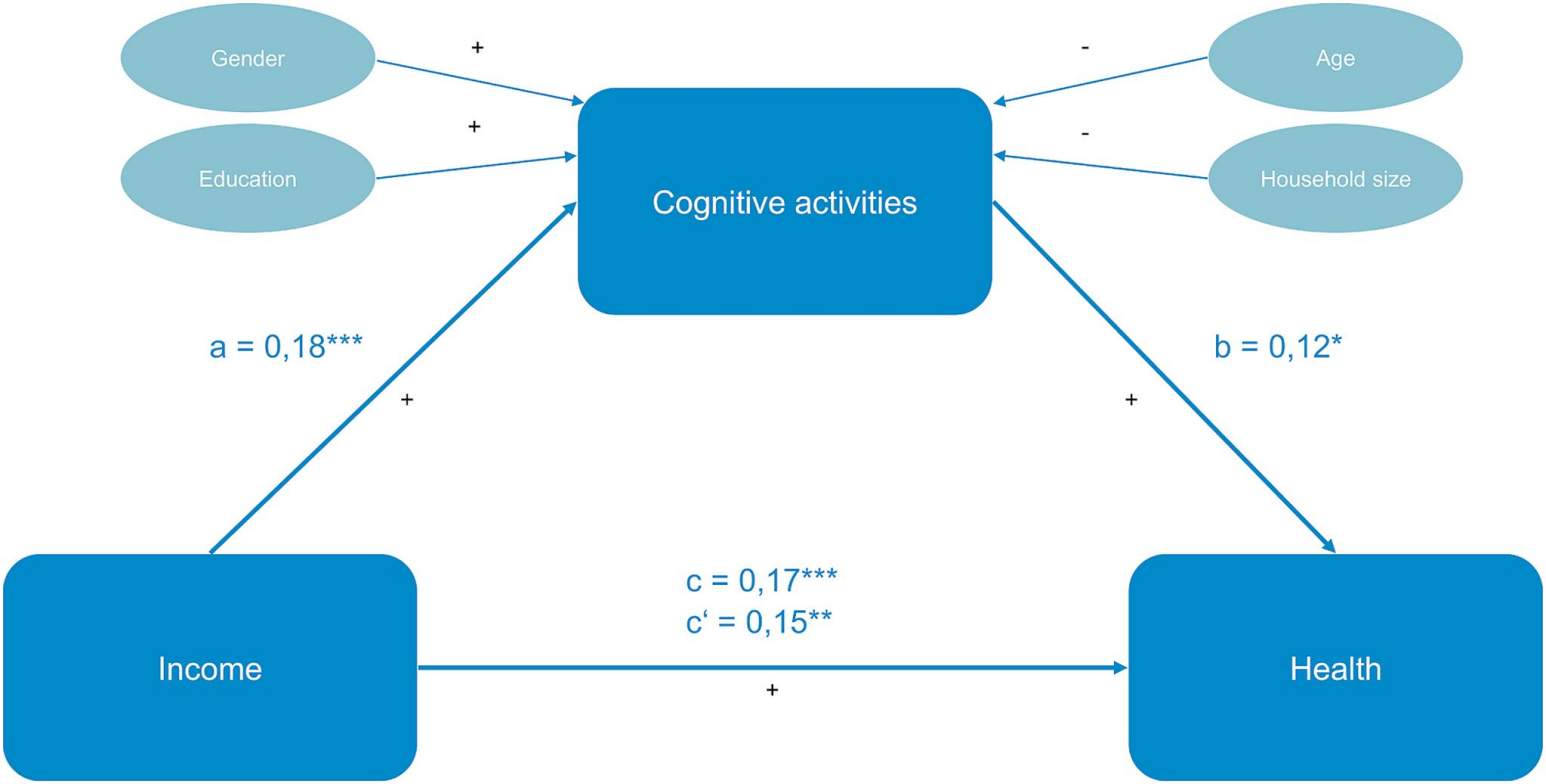
Mediation on household income, cognitive activities, and self-rated health. Significance: *p < 0.05, **p < 0.01, ***p < 0.001. Significant effects are highlighted in green, non-significant in red. + represents a positive effect, − represents a negative effect. c = total effect, c‘= direct effect, a = effect of independent variable on mediator, b = effect of mediator on dependent variable. Only control variables with a significant effect on the mediator variable were included in the figure. Health was measured using self-reported health (self-rated-health, EQ-5D-VAS), income values are given in 100-euro increments based on household income, male gender as reference category.

Cognitive activities partially mediate the association between income and self-rated health, indirect effect *ab* = 0.02; *95% CI*[0.005; 0.045]. This implies that cognitive activities may account for a portion of the effect that household income has on self-rated health. However, there remains a distinct and significant effect between the two variables (see Figure 2). The percentage of the total effect mediated is 12 percent. As shown in Table 3, the frequency of engaging in cognitive activities decreases with increasing age and household size. Conversely, education level appears to have a positive influence. Additionally, women engage in cognitive activities significantly more often than men.

## Discussion

The present study aimed to investigate whether and to what extent various forms of health-related behaviors could explain the association between income and self-rated health. The study was conducted on a sample of individuals aged between 60 and 78 years at the time of data collection, who exhibited an increased risk of developing dementia.

The analyses have yielded, as expected, that higher household income is associated with higher self-rated health. Reasons for this may include material living conditions and income-specific health behaviors (13). This result is consistent with various studies that have found a positive effect of income on health (3, 5, 49–51). The findings indicate that income remains a significant factor even in advanced age and among demographic groups at risk for dementia, as illustrated by the inclusion of individuals with conditions such as obesity and hypertension in the composition of the CAIDE score. However, neither fruit and vegetable consumption nor physical activity could account for this association.

This finding is incongruous with the results of previous studies, which have found a positive effect of income on fruit and vegetable consumption (23–25) and, subsequently, of fruit and vegetable consumption on health (26, 27). Additionally, it contradicts findings suggesting a positive effect of income on physical activity (34, 35). The result of the positive association between physical activity and self-rated health is consistent with various studies (18, 26–28).

The finding that neither fruit and vegetable consumption nor physical activity influenced the association between income and self-rated health contradicts the theoretical assumption that individuals from lower income groups would resort to unhealthier but cheaper food alternatives due to a lack of financial resources. Similarly, it could not be confirmed that they are less physically active because of insufficient financial resources for sports equipment or access to sports courses and events. One plausible explanation for the result could stem from the sample selection. Thus, a CAIDE score of at least 9 points and shared commonalities such as advanced age, lower levels of physical activity, elevated blood pressure and total cholesterol levels already preselects the sample (52). Elevated blood pressure and high cholesterol levels, in turn, may be a consequence of unhealthy diet (26, 27). A portion of physical health, influenced by diet and exercise, may already exhibit burdens in this sample. Additionally, participants in this study, due to this sample selection, may already demonstrate similar behavioral patterns regarding diet and exercise compared to the general population. In the case of a preselected sample, the influence of income on diet and exercise may be underestimated.

However, it was found within the scope of this investigation that social participation mediates the association between income and self-rated health. This relationship could be explained by the fact that some of these social participation opportunities involve monetary costs, which are more challenging for individuals with lower incomes to bear. The results are consistent with various studies that have identified a positive influence of income or socioeconomic status on societal and social participation (5, 32). Additionally, prior research has already found a correlation between higher social engagement and a reduced risk of developing depression (19), dementia (32), or Alzheimer’s disease (33). The present study shows that persons at risk for dementia, including individuals in older age groups with certain health predispositions such as obesity, hypertension, and high cholesterol levels, benefit from social participation. Another notable aspect is that within this investigation, social participation was operationalized beyond social engagement, encompassing various social activities such as traveling or attending events and facilities.

Furthermore, cognitive activity frequency appears to exert a partially mediating effect on the relationship between income and self-rated health. This finding may be attributed to the fact that the acquisition of certain cultural goods and participation in cognitively stimulating events or courses also entail monetary costs that pose a greater financial burden on individuals with lower incomes. It is congruent with previous research that has identified a positive association between higher income and a greater level of participation in cognitive activities (38). Moreover, this finding is consistent with research suggesting a positive effect of cognitive activities on health (20, 33).

The study also found that, compared to men, women consume more fruits and vegetables, participate more regularly in social activities, and engage more frequently in both physical and cognitive activities. The findings contradict the results of Simonson et al., who concluded in their study that women engage in social activities less frequently than men (53). However, it should be noted that the variable of social participation in this study consists only of one-third social engagement, with various other social activities included. Additionally, it contrasts with the findings of Grünheid, which suggest that men engage in sports activities more frequently and for longer durations than women (36). However, they align with previous findings indicating that women consume fruits and vegetables more frequently (*ibid.*) and engage in cognitive activities more often than men (54). These results, showing that women engage more strongly in all four health-promoting activities measured, are surprising given that gender does not significantly influence self-rated health. Furthermore, in subsequent bivariate analyses, no relationship was found between gender and self-rated health. However, they did reveal a higher income for men compared to women. This suggests that despite having lower financial resources on average, women more frequently adopt health-promoting behaviors.

Individuals with higher education levels exhibit greater social participation and are more likely to engage in both physical and cognitive activities. This finding is consistent with the results of Simonson et al., which demonstrate that individuals from higher socioeconomic status groups engage in volunteer activities significantly more frequently, with education being one of three indicators of social status (53). The finding that highly educated individuals engage in more physical activities contradicts the results of Hoebel et al., who found that lower education is associated with higher levels of physical activity (34). However, it should be noted that within the present study, physical activities include deliberate sports participation, while Hoebel et al. distinguish between sports and other physically demanding activities. Another result of this study was that individuals with higher education engage in cognitive activities more frequently than those with lower education. This result aligns with the findings of a study by Wilson et al., which concluded that individuals with more years of education tend to be more cognitively active (54).

An additional outcome is that with increasing age, the frequency of engaging in cognitive activities decreases, which is consistent with the findings of Wilson et al. (54). Moreover, there is an inverse association between the number of household members and the frequency of participation in cognitive activities. One possible explanation could be that individuals with more household members are more socially included, and therefore may have less time or inclination for activities such as puzzles or reading.

### Limitations

Since cross-sectional data were used for the present analyses, it is important to note that this study cannot make definitive conclusions about the direction of the relationships. It is possible that poorer health limits both the opportunities for obtaining higher-paying jobs and people’s ability to engage in health-promoting activities, such as physical exercise. Several studies suggest, however, that the assumption of reverse causality can only explain a small part of health inequalities (55, 56). The more significant explanation appears to be that income influences health behavior, which in turn affects health. In 2002, Mulatu and Schooler examined the relationship between social status and health bidirectionally (56). They found that both factors influence each other, with the influence of social status on health being of greater relevance (*ibid.*).

The sample is preselected based on a CAIDE score of at least 9 points. As discussed in the previous chapter, this preselection may lead to participants exhibiting more homogenous eating and exercise behaviors compared to the general population. Consequently, this could result in an underestimation of the findings.

In the context of the age-as-leveler hypothesis, it should also be considered that there might be another selection bias leading to an underestimation of the influence of income differences in this sample. This hypothesis suggests that in older age groups, individuals with significant health problems or a higher likelihood of premature mortality may participate less frequently in studies (57). Given the assumption that low income could have a negative impact on health, there is a possibility that individuals with low income have already been filtered out, resulting in a more homogeneous sample.

Biases could also arise from the fact that in this study, all variables of interest were collected through self-reports. While this approach offers advantages, such as the ability to include various health aspects in self-rated health, self-reports can be prone to biases due to social desirability or inaccurate self-assessment. Objective measures could provide additional information in future analyses.

### Outlook and implementations

With an explained variance of 3%, a large portion of self-rated health remains unexplained. Future analyses should focus on additional socioeconomic factors, such as employment status, the socioeconomic background of the neighborhood, access to healthcare services, or housing conditions, which may interact with the health behavior of older adults. For example, various studies have identified a significant association between deprived neighborhoods and physical inactivity (58).

Future research could consider including factors such as wealth and homeownership in addition to household income in their analyses. Particularly for studying health disparities among older individuals, the consideration of assets may be relevant to capture financial advantages or disadvantages that accumulate over time (9). The inclusion of homeownership, for example, could help control for rental expenses, which may limit the actual disposable income.

As no mediating effect of fruit and vegetable consumption on the association between income and self-rated health was found in this study, it could be beneficial to incorporate further aspects to assess healthy eating habits. For instance, the DGE advises not only consuming five portions of fruits and vegetables daily but also avoiding fatty foods, consuming dairy products daily, maintaining a varied diet, and regularly opting for whole grain products (59).

To determine where interventions can be particularly effective, it would also be interesting to examine the various forms of societal and social participation more closely. A potential investigation could examine the question of which specific forms of societal and social participation are most frequently selected by low-income groups and which factors influence these decisions. This could help to find out whether differences in social participation between income groups are primarily due to financial barriers or whether people from higher-income brackets also predominantly practice cost-effective social activities. Furthermore, it would be insightful to explore which aspects of social participation have a particularly positive impact on self-rated health in order to derive possible interventions from this.

Since no significant association was found between income and physical activities, it could also be informative to consider the intention behind physical activity. Thus, analogous to Hoebel et al. (34), making a distinction between deliberate engagement in sports and physical activities incidental to necessary tasks, such as movement through household and gardening activities, could be valuable. To determine the intensity of physical or sports activities, it would also be possible to assess not only the frequency but also the duration of the activity. Furthermore, a distinction could be made between cost-free sports activities and those sports that are associated with membership fees for clubs or sports courses. This differentiation would help to further examine the potential influences of financial barriers on physical activity (60).

Another important and interesting finding of our study is that women engage more frequently in health-promoting behaviors despite lower average incomes. This provides insightful information for the implementation for future interventions and should be considered, accordingly. One idea, how men could be involved more in prevention and interventions were surveyed previously (61). Additionally, it could be interesting to take a deeper look at the associations between income, health behavior, and self-rated health across genders. Future studies could address the question of which factors underlie the circumstance that men, despite having a higher average household income than women, exhibit fewer health-promoting behaviors. Moreover, these studies could examine the absence of gender effects on self-rated health despite differences in health behavior.

A factor that could be associated with both income and self-rated health is access to healthcare services. Future analyses could provide further clarity in this regard.

As technology increasingly assumes a significant role in healthcare, it would be valuable to investigate the impact of digital health interventions on low-income older adults. These interventions could potentially facilitate access to healthcare for individuals from lower social strata.

## Conclusion

An important insight garnered from this investigation is the critical role of social participation in elucidating the association between income and self-rated health in an older sample at increased risk. This highlights the imperative to deliberate strategies aimed at dismantling financial barriers and rendering social participation. Another notable finding is the relevance of cognitive activities, which also serve as a conduit for a portion of the impact of income on self-rated health. Hence, health interventions should encompass measures for cognitive stimulation, contemplating the provision of support for learning opportunities and intellectual engagement. It can be concluded that social and cognitive activities mediate the influence of income on self-reported health among individuals over 60 years old who are at increased risk of dementia, while healthy eating and ample exercise do not affect this relationship. Therefore, activities that primarily impact mental and cognitive health may be particularly relevant for this target group. These findings could prompt raise awareness of the mental health of older individuals with low income and considering targeted measures to support this group specifically.

## Data Availability

The data analyzed in this study is subject to the following licenses/restrictions: the datasets cannot be accessed publicly due to privacy constraints. However, researchers can obtain individual participant data, once de-identified, by submitting a well-structured proposal to the AgeWell.de steering committee (contact: steffi.riedel-heller@medizin.uni-leipzig.de). Upon approval of the proposal, access to the datasets can be facilitated. Requests for dataset access should be directed to SR-H, steffi.riedel-heller@medizin.uni-leipzig.de. Requests to access these datasets should be directed to Steffi G. Riedel-Heller, steffi.riedel-heller@medizin.uni-leipzig.de.

## References

[1] Richter M Hurrelmann K. Gesundheitliche Ungleichheit: Grundlagen, Probleme. Perspektiven. Wiesbaden: VS Verlag für Sozialwissenschaften (2009).

[2] Bauer U Bittlingmayer UH Richter M. Health Inequalities: Determinanten und Mechanismen gesundheitlicher Ungleichheit. Wiesbaden: VS Verlag für Sozialwissenschaften (2008).

[3] Wachtler B Rakowitz N. Public Health in Zeiten von Ökonomisierung und zunehmender sozialer Unsicherheit In: Schmidt-Semisch H Schorb F, editors. Public health: Disziplin - praxis - Politik. Wiesbaden: Springer VS (2021). 475–91.

[4] Rathmann K. Bildungsarmut und Gesundheit In: Quenzel G Hurrelmann K, editors. Handbuch Bildungsarmut. Wiesbaden: Springer Fachmedien Wiesbaden (2019). 667–94.

[5] Lampert T Kroll LE. Einfluss der Einkommensposition auf die Gesundheit und Lebenserwartung. Discussion Papers. (2005). 527:1–26.

[6] Hahm S Speerforck S Fleischer T Grabe HJ Beutel M Schomerus G. Die Effekte von Geschlecht, Bildung und Einkommen auf antizipierte Scham bei psychischen Erkrankungen – Ergebnisse einer deutschen Bevölkerungsstudie. Psychiatr Prax. (2020) 47:142–7. doi: 10.1055/a-1081-7614, PMID: 31952086

[7] Binnewies C Sonnentag S. Arbeitsbedingungen, Gesundheit und Arbeitsleistung In: Leidig S, editor. Stress im Erwerbsleben: Perspektiven eines integrativen Gesundheitsmanagements. Lengerich: Pabst Science Publishers (2006). 47–69.

[8] Reibling N Jutz R. Energiearmut und Gesundheit. Die Bedeutung von Wohnbedingungen für die soziale Ungleichheit im Gesundheitszustand In: Großmann K Schaffrin A Smigiel C, editors. Energie und soziale Ungleichheit: Zur gesellschaftlichen Dimension der Energiewende in Deutschland und Europa. Wiesbaden: Springer VS (2017). 157–84.

[9] Knesebeck Ov. Soziale Ungleichheit und Gesundheit im Alter – Klassische oder alternative Statusindikatoren? Z Gerontol Geriatr. (2002) 35:224–31. doi: 10.1007/s00391-002-0048-y 12219707

[10] Livingston G Sommerlad A Orgeta V Costafreda SG Huntley J Ames D. Dementia prevention, intervention, and care. Lancet. (2017) 390:2673–734. doi: 10.1016/S0140-6736(17)31363-6 28735855

[11] Anttila T Helkala E-L Kivipelto M Hallikainen M Alhainen K Heinonen H. Midlife income, occupation, APOE status, and dementia: a population-based study. Neurology. (2002) 59:887–93. doi: 10.1212/WNL.59.6.887, PMID: 12297572

[12] Scazufca M Almeida OP Menezes PR. The role of literacy, occupation and income in dementia prevention: the São Paulo Ageing & Health Study (SPAH). Int Psychogeriatr. (2010) 22:1209–15. doi: 10.1017/S1041610210001213, PMID: 20678301

[13] Bartley M. Health inequality: An introduction to concepts, theories and methods. Hoboken: John Wiley & Sons (2016).

[14] Carstensen J. Die Messung von Gesundheit In: Kriwy P Jungbauer-Gans M, editors. Handbuch Gesundheitssoziologie. Wiesbaden: Springer Fachmedien Wiesbaden (2020). 51–70.

[15] De Bruin A. Health interview surveys: towards international harmonization of methods and instruments. Copenhagen: World Health Organization Regional Office for Europe. (WHO regional publications European series / WHO, Regional Office for Europe (1996). 8857196

[16] Benyamini Y Blumstein T Murad H Lerner-Geva L. Changes over time from baseline poor self-rated health: for whom does poor self-rated health not predict mortality? Psychol Health. (2011) 26:1446–62. doi: 10.1080/08870446.2011.559231 22011289

[17] Lazarevič P. Was misst self-rated health? Wiesbaden: Springer Fachmedien Wiesbaden (2019).

[18] Norton S Matthiews FE Barnes DE Yaffe K Brayne C. Potential for primary prevention of Alzheimer’s disease: an analysis of population-based data. Lancet Neurol. (2014) 13:788–94. doi: 10.1016/S1474-4422(14)70136-X, PMID: 25030513

[19] Humpert S. Gender differences in life satisfaction and social participation. Int J Econ Sci Appl Res. (2013) 6:123–42.

[20] Wilson RS Segawa E Boyle PA Bennett DA. Influence of late-life cognitive activity on cognitive health. Neurology. (2012) 78:1123–9. doi: 10.1212/WNL.0b013e31824f8c03, PMID: 22491864 PMC3320053

[21] Duffner LA DeJong NR Jansen JF Backes WH De VM Deckers K. Associations between social health factors, cognitive activity and neurostructural markers for brain health – a systematic literature review and meta-analysis. Ageing Res Rev. (2023) 89:101986. doi: 10.1016/j.arr.2023.101986, PMID: 37356551

[22] Mensink G Truthmann J Rabenberg M Heidemann C Haftenberger M Schienkiewitz A. Obst- und Gemüsekonsum in Deutschland: Ergebnisse der Studie zur Gesundheit Erwachsener in Deutschland (DEGS1). Bundesgesundheitsbl. (2013) 56:779–85. doi: 10.1007/s00103-012-1651-8, PMID: 23703498

[23] Goryńska-Goldmann E Murawska A Balcerowska-Czerniak G. Consumer profiles of sustainable fruit and vegetable consumption in the European Union. Sustain For. (2023) 15:15512. doi: 10.3390/su152115512

[24] Lallukka T Pitkäniemi J Rahkonen O Roos E Laaksonen M Lahelma E. The association of income with fresh fruit and vegetable consumption at different levels of education. Eur J Clin Nutr. (2010) 64:324–7. doi: 10.1038/ejcn.2009.155, PMID: 20087380

[25] Stea TH Nordheim O Bere E Stornes P Eikemo TA. Fruit and vegetable consumption in Europe according to gender, educational attainment and regional affiliation - a cross-sectional study in 21 European countries. PLoS One. (2020) 15:e0232521. doi: 10.1371/journal.pone.0232521, PMID: 32401798 PMC7219700

[26] Atzpodien K Bergmann E Bertz J Busch M Eis D Ellert U. 20 Jahre nach dem Fall der Mauer: Wie hat sich die Gesundheit in Deutschland entwickelt?. (2009). Berlin: Robert Koch-Institut.

[27] Löllgen H Löllgen R. Genetics, genetic testing and sports: aspects from sports cardiology. Genomics Soc Policy. (2012) 8:1–16. doi: 10.1186/1746-5354-8-1-32

[28] Wollesen B Dahlke B Stehr G. Auswirkungen der Armut auf die Gesundheit von Senioren im Bezirk Altona: 2. Altonaer Gesundheitsbericht, vol. 76. Hamburg: Hamburg.de (2014). 10.1055/s-0035-1555947 26335656

[29] Schlegel-Matthies K. Die Bedeutung von Ressourcen für Lebensqualität und gesellschaftliche Teilhabe. HiBiFo–Haushalt in Bildung und. Forschung. (2022) 11:3–24. doi: 10.3224/hibifo.v11i1.01

[30] Buchholz M Zöllinger I Thyrian JR Luppa M Zülke A Döhring J. Factors associated with lower social activity in German older adults at risk of dementia – a cross-sectional analysis. J Alzheimers Dis. (2024) 98:1443–55. doi: 10.3233/JAD-231226, PMID: 38607756

[31] Ahmad K Hafeez M. Factors affecting social participation of elderly people: a study in Lahore. J Anim Plant Sci. (2011) 21:283–9.

[32] Kurz A. Psychosoziale Interventionen bei Demenz. Nervenarzt. (2013) 84:93–105. doi: 10.1007/s00115-012-3655-x 23306213

[33] Lindstrom H Fritsch T Petot G Smyth K Chen C Debanne S. The relationships between television viewing in midlife and the development of Alzheimer’s disease in a case-control study. Brain Cogn. (2005) 58:157–65. doi: 10.1016/j.bandc.2004.09.020, PMID: 15919546

[34] Hoebel J Finger JD Kuntz B Lampert T. Sozioökonomische Unterschiede in der körperlich-sportlichen Aktivität von Erwerbstätigen im mittleren Lebensalter. Bundesgesundheitsblatt. (2016) 59:188–96. doi: 10.1007/s00103-015-2278-3, PMID: 26620205

[35] Thibaut E Eakins J Willem A Scheerder J. Financial barriers for sports consumption: the dynamics of the income-expenditure relation. Int J. (2020) 10:245–61. doi: 10.1108/SBM-04-2019-0026

[36] Grünheid E. Einflüsse der Einkommenslage auf Gesundheit und Gesundheitsverhalten: Ergebnisse des Lebenserwartungssurveys des BiB. Wiesbaden: Bundesinstitut für Bevölkerungsforschung (BIB) (2004).

[37] Netz Y Wu M-J Becker BJ Tenenbaum G. Physical activity and psychological well-being in advanced age: a Meta-analysis of intervention studies. Psychol Aging. (2005) 20:272–84. doi: 10.1037/0882-7974.20.2.272 16029091

[38] Litwin H Schwartz E Damri N. Cognitively stimulating leisure activity and subsequent cognitive function: a SHARE-based analysis. The Gerontologist. (2017) 57:940–8. doi: 10.1093/geront/gnw084, PMID: 27117305 PMC5881687

[39] Röhr S Zülke A Luppa M Brettschneider C Weißenborn M Kühne F. Recruitment and baseline characteristics of participants in the AgeWell.de study—a pragmatic cluster-randomized controlled lifestyle trial against cognitive decline. Int J Environ Res Public. (2021) 18:408–422. doi: 10.3390/ijerph18020408 PMC7825589 33430189

[40] Zülke A Luck T Pabst A Hoffmann W Thyrian JR Gensichen J. AgeWell.de – study protocol of a pragmatic multi-center cluster-randomized controlled prevention trial against cognitive decline in older primary care patients. BMC Geriatr. (2019) 19:203. doi: 10.1186/s12877-019-1212-1, PMID: 31370792 PMC6670136

[41] The EuroQol Group. EuroQol - a new facility for the measurement of health-related quality of life. Health Policy. (1990) 16:199–208. doi: 10.1016/0168-8510(90)90421-9 10109801

[42] Schneider S Konzeptualisierung D. Erhebung und Kodierung von Bildung in nationalen und internationalen Umfragen (Version 1.1): GESIS -. Mannheim: Leibniz-Institut für Sozialwissenschaften (2016).

[43] Verghese J Lipton RB Katz MJ Hall CB Derby CA Kuslansky G. Leisure activities and the risk of dementia in the elderly. N Engl J Med. (2003) 348:2508–16. doi: 10.1056/NEJMoa022252 12815136

[44] Bortz J Döring N. Forschungsmethoden und Evaluation für Human- und Sozialwissenschaftler. Heidelberg: Springer Medizin Verlag (2006).

[45] Weise J. Methodik der Datenauswertung In: Weise J, editor. Planung und Steuerung von Innovationsprojekten. Wiesbaden: DUV (2008). 169–218.

[46] Cohen J. A power primer. Psychol Bull. (1992) 112:155–9. doi: 10.1037/0033-2909.112.1.155, PMID: 19565683

[47] Schneider M. Datenanalyse für Naturwissenschaftler, Mediziner und Ingenieure. Berlin: Springer Spektrum (2020).

[48] Hayes AF. Introduction to mediation, moderation, and conditional process analysis: a regression-based approach, vol. 2. New York: Guilford Press (2018).

[49] Lichtenberger V. Der Einfluss des Einkommens auf die Gesundheit Statistisches Monatsheft Baden-Württemberg (2013) 7, 21–26. Available at: https://nbn-resolving.org/urn:nbn:de:0168-ssoar-420804.

[50] Livingston G Huntley J Sommerlad A Ames D Ballard C Banerjee S. Dementia prevention, intervention, and care: 2020 report of the lancet commission. Lancet. (2020) 396:413–46. doi: 10.1016/S0140-6736(20)30367-6, PMID: 32738937 PMC7392084

[51] Helmert U Mielck A Shea S. Poverty and health in West Germany. Soz Praventivmed. (1997) 42:276–85. doi: 10.1007/BF01592324 9403948

[52] Rundek T Gardener H Dias Saporta AS Loewenstein DA Duara R Wright CB. Global vascular risk score and CAIDE dementia risk score predict cognitive function in NOMAS. J Alzheimers Dis. (2020) 73:1221–31. doi: 10.3233/JAD-190925, PMID: 31884476 PMC7880168

[53] Simonson J Hagen C Vogel C Motel-Klingebiel A. Ungleichheit sozialer Teilhabe im Alter. Z Gerontol Geriatr. (2013) 46:410–6. doi: 10.1007/s00391-013-0498-4 23864320

[54] Wilson RS Bennett DA Beckett LA Morris MC Gilley DW Bienias JL. Cognitive activity in older persons from a geographically defined population. J Gerontol Psychol Sci. (1999) 54B:P155–60. doi: 10.1093/geronb/54B.3.P155 10363036

[55] Wilkinson RG. Gesundheit, Hierarchie und soziale Angst In: Insider und Outsider Graz: Denkwerkstätte (2004) 119–43.

[56] Mulatu MS Schooler C. Causal connections between socio-economic status and health: reciprocal effects and mediating mechanisms. J Health Soc Behav. (2002) 43:22–41. doi: 10.2307/3090243, PMID: 11949195

[57] House JS Lepkowski JM Kinney AM Mero RP Kessler RC Herzog AR. The social stratification of aging and health. J Health Soc Behav. (1994) 35:213–34. doi: 10.2307/2137277 7983335

[58] Algren MH Bak CK Berg-Beckhoff G Andersen PT. Health-risk behaviour in deprived neighbourhoods compared with non-deprived neighbourhoods: a systematic literature review of quantitative observational studies. PLoS One. (2015) 10:e0139297. doi: 10.1371/journal.pone.0139297, PMID: 26506251 PMC4624433

[59] Jungvogel A Wendt I Schäbethal K Leschik-Bonnet E Oberritter H. Überarbeitet: Die 10 Regeln der DGE. Ernährungs Umschau. (2013) 11:644–5.

[60] Müller B Kropp P Cardona MI Michalowsky B van den Berg N Teipel S. Types of leisure time physical activities (LTPA) of community-dwelling persons who have been screened positive for dementia. BMC Geriatr. (2021) 21:270. doi: 10.1186/s12877-021-02201-1, PMID: 33892624 PMC8063325

[61] Wittmann FG Zülke A Schultz A Claus M Röhr S Luppa M. Beneficial and impeding factors for the implementation of health-promoting lifestyle interventions-a gender-specific focus group study. Int J Environ Res Public Health. (2023) 20:3520–3534. doi: 10.3390/ijerph20043520, PMID: 36834215 PMC9967898

